# Trajectories of BMI before and after diagnosis of type 2 diabetes in a real-world population

**DOI:** 10.1007/s00125-024-06217-1

**Published:** 2024-07-05

**Authors:** Louise A. Donnelly, Rory J. McCrimmon, Ewan R. Pearson

**Affiliations:** 1 Division of Population Health and Genomics, School of Medicine, University of Dundee, Dundee, UK; 2 Division of Systems Medicine, School of Medicine, University of Dundee, Dundee, UK

**Keywords:** BMI trajectories, Electronic medical records, Longitudinal analysis, Obesity, Observational, Real-world data, Stratification, Type 2 diabetes, Weight trajectories

## Abstract

**Aims/hypothesis:**

Few studies have examined the clinical characteristics associated with changes in weight before and after diagnosis of type 2 diabetes. Using a large real-world cohort, we derived trajectories of BMI before and after diabetes diagnosis, and examined the clinical characteristics associated with these trajectories, including assessing the impact of pre-diagnosis weight change on post-diagnosis weight change.

**Methods:**

We performed an observational cohort study using electronic medical records from individuals in the Scottish Care Information Diabetes Collaboration database. Two trajectories were calculated, based on observed BMI measurements between 3 years and 6 months before diagnosis and between 1 and 5 years after diagnosis. In the post-diagnosis trajectory, each BMI measurement was time-dependently adjusted for the effects of diabetes medications and HbA_1c_ change.

**Results:**

A total of 2736 individuals were included in the study. There was a pattern of pre-diagnosis weight gain, with 1944 individuals (71%) gaining weight overall, and 875 (32%) gaining more than 0.5 kg/m^2^ per year. This was followed by a pattern of weight loss after diagnosis, with 1722 individuals (63%) losing weight. Younger age and greater social deprivation were associated with increased weight gain before diagnosis. Pre-diagnosis weight change was unrelated to post-diagnosis weight change, but post-diagnosis weight loss was associated with older age, female sex, higher BMI, higher HbA_1c_ and weight gain during the peri-diagnosis period. When considering the peri-diagnostic period (defined as from 6 months before to 12 months after diagnosis), we identified 986 (36%) individuals who had a high HbA_1c_ at diagnosis but who lost weight rapidly and were most aggressively treated at 1 year; this subgroup had the best glycaemic control at 5 years.

**Conclusions/interpretation:**

Average weight increases before diagnosis and decreases after diagnosis; however, there were significant differences across the population in terms of weight changes. Younger individuals gained weight pre-diagnosis, but, in older individuals, type 2 diabetes is less associated with weight gain, consistent with other drivers for diabetes aetiology in older adults. We have identified a substantial group of individuals who have a rapid deterioration in glycaemic control, together with weight loss, around the time of diagnosis, and who subsequently stabilise, suggesting that a high HbA_1c_ at diagnosis is not inevitably associated with a poor outcome and may be driven by reversible glucose toxicity.

**Graphical Abstract:**

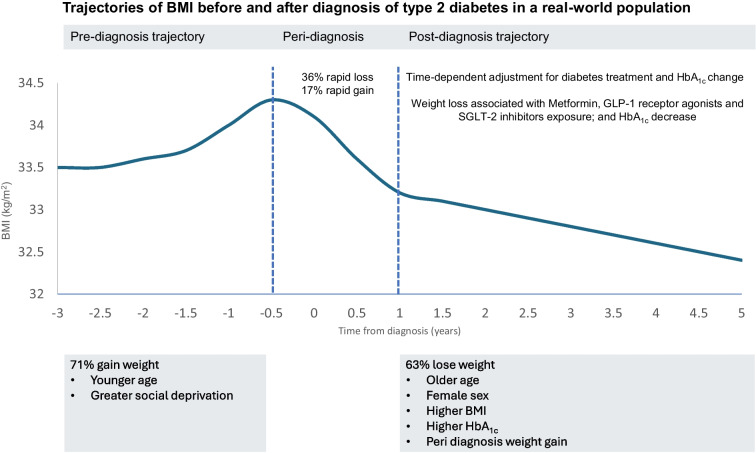

**Supplementary Information:**

The online version of this article (10.1007/s00125-024-06217-1) contains peer-reviewed but unedited supplementary material.


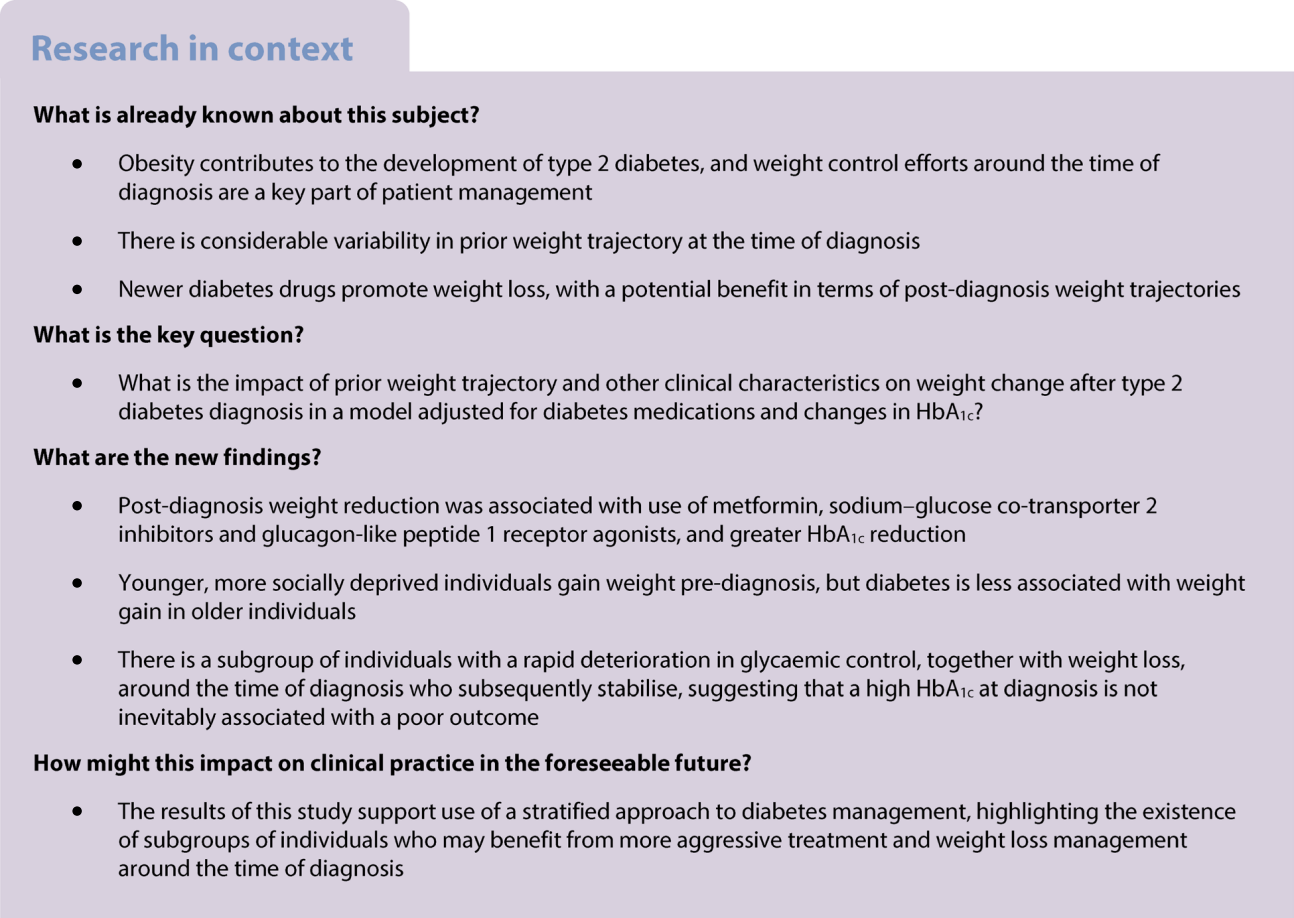


## Introduction

Many epidemiological studies have demonstrated the association between obesity and risk of development of type 2 diabetes [1], and shown that weight reduction is a key component of type 2 diabetes management. The period around diagnosis is an important treatment window in clinical practice, as weight loss produces short-term improvements in glycaemic control [2, 3]. Additionally, it has been reported that patients may be more motivated to lose weight directly after being diagnosed with type 2 diabetes [4].

There is considerable variability between patients at the time of type 2 diabetes diagnosis in terms of weight, weight gain and duration of being overweight or obese [5–7]. To our knowledge, only two studies have explored the association between changes in weight before and after diagnosis [8, 9]. Furthermore, only one of these studies specifically investigated the influence of an individual’s history of weight change before diagnosis on the observed weight changes after diagnosis [8]. However, this study comprised only 885 individuals and relied on self-reported BMI measurements to establish weight history.

Previous studies have investigated long-term trajectories of BMI before diagnosis of type 2 diabetes [10, 11]. However, limited data is available from observational studies on longitudinal weight change after diagnosis of type 2 diabetes. A large study using the national Scottish Diabetes Register followed individuals diagnosed between 2002 and 2006 for 2 years after diagnosis [12], and identified groups who may benefit most from weight loss management (men, younger individuals, and those with lower BMI at diagnosis and higher social deprivation). However, this study did not incorporate a measure of weight history before diagnosis, and few individuals were receiving newer diabetes medications.

Modelling weight change after type 2 diabetes diagnosis is challenging, as it is difficult to separate the effects of glycaemic control and pharmacological interventions from those of weight change [13]. We have previously developed a novel approach for modelling the rate of glycaemic deterioration in observational data across multiple drug combinations [14]. We adapt this approach in the current study to time-dependently adjust each individual’s observed BMI measures for the effects of diabetes medications and changes in HbA_1c_.

The aims of this study were to use a large population-based cohort with type 2 diabetes and routinely collected repeated measurements of BMI to derive trajectories of weight change before and after diagnosis of type 2 diabetes, and to investigate clinical characteristics associated with these trajectories and the impact of diabetes drugs and HbA_1c_ changes. Of particular interest was the extent to which weight change before diagnosis influences weight change after. Gaining insight into the relationship between prior weight trajectory (alongside other key clinical patient characteristics at diagnosis) and longitudinal weight change after diagnosis may enable a more stratified approach to diabetes management, highlighting subgroups of individuals who may benefit from more aggressive treatment and weight loss management around the time of diagnosis.

## Methods

An observational cohort study was performed using comprehensive electronic medical records from all individuals with type 2 diabetes resident in Tayside and Fife, Scotland. Data were collected and integrated by the Health Informatics Centre, University of Dundee. Record linkage was through the community health index number provided to all individuals in Scotland when they register with a primary healthcare provider, which has been used continuously for all National Health Service clinical activity over the past 30 years.

The Scottish Care Information – Diabetes Collaboration database provided information on type of diabetes, diagnosis date and, from calendar year 2000 onwards, clinically measured BMI. The demography database provided information on age, sex, health board (Tayside or Fife) and social deprivation (residential areas are categorised and ranked into health board quintiles based on an individual’s postcode). Ethnicity data were not collected. Scotland is a predominantly white population (87% according to Scotland’s Census 2022 [15]), and ethnicity was therefore not considered to be relevant to this study. The biochemistry database was used to obtain measurements of HbA_1c_ from 1995 onwards. Finally, the community-dispensed prescribing database, which contains detailed information on all fulfilled prescriptions prescribing in Tayside from 1995 onwards and Fife from 2009 onwards, was used to identify diabetes medications.

### Study design

**Fig. 1:**
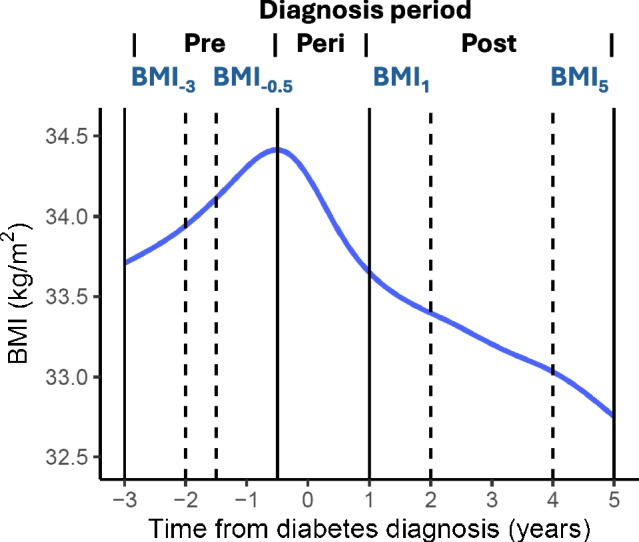
Pattern of all BMI measurements during the 8-year study period for all eligible individuals, with a smooth line fitted through the population mean. The solid vertical lines show the split of the study period into pre-, peri- and post-diagnosis periods, and the dotted vertical lines indicate the timing of the required BMI measurements: BMI_−3_ (closest to 3 years before diagnosis in the −3 to −2 year window), BMI_−0.5_ (closest to 6 months before diagnosis in the −18 to −6 month window), BMI_1_ (closest to 1 year after diagnosis in the 1 to 2 year window) and BMI_5_ (closest to 5 years in the 4 to 5 year window)

We defined the study period as from 3 years before diabetes diagnosis to 5 years after diabetes diagnosis. All BMI measurements during this time were considered; however, to allow separate pre- and post-diagnosis trajectories to be derived, there was a minimum data requirement of four measurements per individual within specific time windows: BMI measurements between 3 and 2 years and 1 and 0 years before diagnosis to derive the pre-diagnosis trajectory, and BMI measurements between 0 and 1 years and 4 and 5 years after diagnosis to derive a post-diagnosis trajectory. Figure 1 shows the pattern of all BMI measurements from the eligible individuals during the 8-year study period. There was a pattern of significant weight loss between 6 months before and 1 year after diagnosis, therefore we did not include this ‘peri-diagnosis’ period in the pre- or post-diagnosis trajectories. Our revised study design required four BMI measurements defined as: BMI_−3_ (that closest to 3 years before diagnosis in a −3 to −2 year window), BMI_−0.5_ (that closest to 6 months before diagnosis in a −18 to −6 month window), BMI_1_ (that closest to 1 year after diagnosis in a 1 to 2 year window) and BMI_5_ (that closest to 5 years after diagnosis in a 4 to 5 year window).

### Study population

To ensure sufficient electronic medical record coverage and avoid the impact of COVID-19 on our data, the study period was defined as 1 January 2000 until 31 December 2019. Individuals with a clinical diagnosis of type 2 diabetes between 1 January 2003 and 31 December 2014, diagnosed at ≥ 35 years of age, with a BMI_1_ ≥ 25 kg/m^2^ and an HbA_1c_ measurement between 1 and 2 years after diagnosis were eligible. A flow chart of the study population derivation is provided in electronic supplementary material (ESM) Fig. 1.

### Pre-diagnosis BMI trajectory

A model was derived for each individual’s weight change based on observed BMI measurements between 3 years and 6 months before diabetes diagnosis (BMI_−3_ and BMI_−0.5_). Covariates in the model were age at BMI_−3_ (split into bands: <50, 50 to <60, 60 to <70 and ≥70 years), sex, social deprivation coded as 1 (most deprived) to 5 (least deprived), calendar year at BMI_−3_ (split into approximate quartiles: <2006, 2006 and 2007, 2008 and 2009, 2010 and 2011) and BMI_−3_ (split into categories: <30, 30 to <35, 35 to <40 and ≥40 kg/m^2^).

### Post-diagnosis BMI trajectory

A model was derived for each individual’s weight change based on observed BMI measurements between 1 year and 5 years after diabetes diagnosis (BMI_1_ and BMI_5_). BMI measurements were time-dependently adjusted for diabetes treatment and HbA_1c_ change. BMI measurements obtained before medications were started were used as the reference group. Treatments were grouped by drug class: metformin, sulfonylureas, thiazolidinediones, dipeptidyl peptidase 4 inhibitors (DPP4i), glucagon-like peptide 1 receptor agonists (GLP-1RA), sodium–glucose co-transporter 2 inhibitors (SGLT2i) and insulin; and further grouped by insulin (including any other diabetes treatment), monotherapy and various dual and triple therapy combinations. HbA_1c_ change was expressed as the percentage change from the first HbA_1c_ measurement after 1 year from diagnosis, and categorised into five groups: no change (reference group), >0 to <10% decrease, ≥10% decrease, >0 to <10% increase and ≥10% increase.

Covariates in the model were age at diagnosis (split into bands: <50, 50 to <60, 60 to <70 and ≥70 years), sex, social deprivation coded as 1 (most deprived) to 5 (least deprived), calendar year of diagnosis (split into approximate quartiles: 2003 to 2008, 2009 and 2010, 2011 and 2012, 2013 and 2014), BMI_1_ (split into categories: 25 to <30, 30 to <35, 35 to <40 and ≥40 kg/m^2^) and HbA_1c_ (measured closest to 1 year after diagnosis but between 1 and 2 years) split into approximate quartiles (<43, 43 to <48, 48 to <54 and ≥54 mmol/mol; <6.1, 6.1 to <6.5, 6.5 to <7.1 and ≥7.1%).

To model the effect of prior weight change on the post-diagnosis BMI trajectory, each individual’s pre-diagnosis trajectory was included as a covariate. In addition, as BMI measurements in the peri-diagnosis period were not included in the pre- or post-diagnosis trajectories, we defined a peri-diagnosis weight change, calculated as (BMI_1_ − BMI_−0.5_)/time (in years) between measurements.

The peri-diagnosis weight change and the pre-diagnosis trajectory were split into seven categories of BMI change: rapid loss (>0.5 kg/m^2^/year), moderate loss (>0.3 to 0.5 kg/m^2^/year), slow loss (>0.1 to 0.3 kg/m^2^/year), stable (±0.1 kg/m^2^/year), slow gain (>0.1 to 0.3 kg/m^2^/year), moderate gain (>0.3 to 0.5 kg/m^2^/year), rapid gain (>0.5 kg/m^2^/year).

### Statistical analysis

To calculate pre- and post-diagnosis trajectories, we applied a linear mixed-effects model with both a fixed and random intercept and slope. To describe the covariance structure among the errors, we used the ‘continuous time/continuous space’ spatial data covariance structure provided within the PROC MIXED procedure in SAS version 9.4 (SAS Institute, USA). After the initial model was fitted, the Studentised residuals were examined, and any BMI measurements more than three standard deviations from the mean were removed as outliers (approximately 1.2% of all measurements) and the model was refitted.

Pre- and post-diagnosis BMI trajectories were calculated by adding together each individual’s random slope with the population average (fixed) slope. These trajectories may be interpreted as BMI change per year, indicating the direction and magnitude of the change in an individual’s BMI over the specified time period.

The models were expanded to include covariates. To model the effect of each covariate on weight change, an interaction term between the covariate and time was included. In addition, the post-diagnosis BMI trajectory models were adjusted for diabetes treatment and changes in HbA_1c_ over time, fitted as fixed effects. We fitted univariable models, and, after a full exploratory analysis, selected a multivariable model that included all significant covariates and any important confounders.

**Fig. 2:**
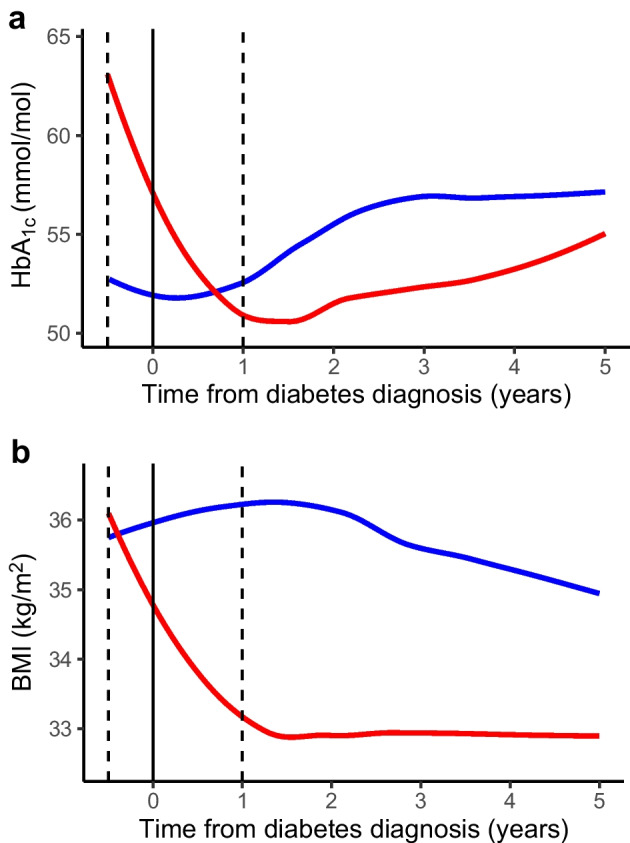
Patterns of HbA_1c_ and BMI measurements. (**a**) Pattern of HbA_1c_ measurements during the peri- and post-diagnosis periods for individuals with rapid weight loss (red line) or rapid weight gain (blue line) during the peri-diagnosis period. (**b**) Pattern of BMI measurements during the peri- and post-diagnosis periods for individuals with rapid weight loss (red line) or rapid weight gain (blue line) during the peri-diagnosis period. The curves are fitted through the population mean. The vertical lines highlight the time of diagnosis (solid line) and the peri-diagnosis window (dotted lines)

We used the PROC SGPLOT procedure in SAS to produce scatterplots of BMI and HbA_1c_ measurements over time (Figs 1 and 2a,b). To explore overall patterns, we used the LOESS statement to fit a smoothed line through the population mean. These plots are intended as a simple visual representation of the data, with all measurements treated as independent, and are not the output from the mixed models.

A *p* value <0.05 was considered statistically significant in all analyses.

## Results

After restriction of the study to individuals with sufficient data to allow pre- and post-diagnosis BMI trajectories to be derived, 2736 individuals were included in the study (ESM Fig. 1). A comparison of the characteristics at 1 year from diabetes diagnosis for individuals who were included vs those who were excluded is shown in ESM Table 1; the comparison is restricted to individuals with BMI ≥25 kg/m^2^ for whom an HbA_1c_ measurement was obtained between 1 and 2 years after diagnosis. The study population were older than the excluded patients (65.6±10.1 vs 61.1±11.4 years, mean ± SD), with lower HbA_1c_ (50.6±12.5 vs 53.8±14.8 mmol/mol/6.8±1.1 vs 7.1±1.4%), more likely to be treated through diet alone (64.8% vs 50.1%) and more recently diagnosed (51.9% from 2011 onwards vs 46.0%). There was no significant difference between groups for sex (43% of the study population were female), BMI (33±5.9 kg/m^2^ for the study population) or social deprivation.

### BMI trajectory before diabetes diagnosis

During the 30-month study period before diabetes diagnosis, the median (IQR) number of BMI measurements per individual was 3 (2–4). The median (IQR) BMI change was 0.25 kg/m^2^ per year (−0.06 to 0.63). Overall, 1944 individuals (71.1%) gained weight, with 875 (32.0%) gaining more than 0.5 kg/m^2^ per year, compared with 195 (7.1%) who lost more than 0.5 kg/m^2^ per year.

#### Clinical characteristics associated with weight change

We expanded the model to include clinical covariates. The results for the overall models are presented in Table 1. In the univariable analyses, younger age, higher social deprivation and earlier calendar year were associated with weight gain before diagnosis of diabetes. In the multivariable model, younger age remained strongly associated with weight gain: individuals younger than 50 years gained on average (95% CI) 0.38 kg/m^2^ per year (95% CI 0.24, 0.52) more than those 70 years or older. In addition, the two most socially deprived quintiles gained on average 0.15 kg/m^2^ per year (95% CI 0.02, 0.27) and 0.15 kg/m^2^ per year (95% CI 0.02, 0.28) more than the least deprived quintile, and individuals in calendar years 2008 and 2009, 30 months prior to diabetes diagnosis, gained on average 0.15 kg/m^2^ per year (95% CI 0.03, 0.26) more than those in 2010 and 2011.

**Table 1:** Differences in estimated BMI change before diabetes diagnosis

Variable	*n*	Univariable analysis	Multivariable analysis
Sex			
Female	1181	0.06 (−0.02, 0.14)	
Male	1555	Reference	
Age (years)			
<50	323	0.40 (0.26, 0.54)	0.38 (0.24, 0.52)
50 to <60	664	0.29 (0.17, 0.40)	0.27 (0.16, 0.38)
60 to <70	1009	0.17 (0.07, 0.27)	0.16 (0.05, 0.26)
≥70	740	Reference	Reference
BMI (kg/m^2^)			
<30	852	Reference	
30 to <35	1045	−0.08 (−0.18, 0.02)	
35 to <40	516	−0.06 (−0.17, 0.06)	
≥40	323	−0.06 (−0.20, 0.08)	
Social deprivation			
1 (most deprived)	598	0.18 (0.05, 0.31)	0.15 (0.02, 0.27)
2	626	0.16 (0.03, 0.29)	0.15 (0.02, 0.28)
3	502	0.12 (−0.02, 0.25)	0.11 (−0.03, 0.24)
4	465	−0.03 (−0.17, 0.11)	−0.03 (−0.17, 0.10)
5 (least deprived)	485	Reference	Reference
Missing	60	0.19 (−0.11, 0.48)	0.15 (−0.14, 0.45)
Calendar year			
<2006	676	0.11 (0.0001, 0.23)	0.11 (−0.01, 0.22)
2006 and 2007	640	0.10 (−0.02, 0.21)	0.09 (−0.03, 0.21)
2008 and 2009	733	0.16 (0.04, 0.27)	0.15 (0.03, 0.26)
2010 and 2011	687	Reference	Reference

For the univariable analysis, values are unadjusted coefficients (95% CI). Units are BMI change (kg/m^2^) per year (over a 30-month study period). For the multivariable analysis, values are adjusted coefficients (95% CI). Units are BMI change (kg/m^2^) per year (over a 30-month study period), adjusted for age, calendar year and social deprivation at 3 years before diagnosis. Values are expressed as the difference in BMI change between the study group and the reference group. A positive value indicates weight gain and a negative value indicates weight loss compared with the reference group

### BMI trajectory after diabetes diagnosis

During the 4-year study period after diabetes diagnosis, the median (IQR) number of BMI measurements per individual was 7 (5–9). The median (IQR) BMI change was −0.14 kg/m^2^ per year (−0.46 to 0.14). Overall, 1722 individuals (62.9%) lost weight, with 622 (22.7%) losing more than 0.5 kg/m^2^ per year, compared with 211 (7.7%) who gained more than 0.5 kg/m^2^ per year.

#### Linear mixed model-derived associations

The linear mixed-effects model was time-dependently adjusted for diabetes treatment. The estimates for the most commonly prescribed treatments are presented in ESM Table 2. These represent the model-derived estimates for BMI change associated with a particular treatment combination compared with no treatment. A total of 53.7% of BMI measurements were diet-treated alone. Metformin monotherapy was the most common treatment (29.2% of measurements) and was associated with a reduction in BMI of 0.32 kg/m^2^ on average (95% CI 0.24, 0.40) compared with no treatment, whereas insulin, sulfonylureas and thiazolidinediones were associated with increases in BMI of 0.29 kg/m^2^ (0.01, 0.58), 0.31 kg/m^2^ (0.14, 0.48) and 1.22 kg/m^2^ (0.76, 1.68), respectively (means and 95% CI). In addition, metformin in combination with SGLT2i, GLP-1RA or DPP4i was associated with a reduction in BMI of 1.04 kg/m^2^ (0.71, 1.37), 0.79 kg/m^2^ (0.34, 1.24) and 0.39 kg/m^2^ (0.19, 0.59), respectively.

The model was also adjusted for %HbA_1c_ change over time. The estimates are presented in ESM Table 3. Compared with no change, an HbA_1c_ decrease of ≥10% was associated with a mean reduction in BMI of 0.43 kg/m^2^ (95% CI 0.36, 0.50) and an increase of ≥10% was associated with a mean increase in BMI of 0.48 kg/m^2^ (95% CI 0.42, 0.53).

### Clinical characteristics associated with weight change

We expanded the model to include clinical covariates. The results for the overall models are presented in Table 2. In the univariable analysis, clinical characteristics associated with weight loss after 1 year post-diagnosis were female sex, older age, higher BMI, higher HbA_1c_ and gaining weight during the peri-diagnosis period.

**Table 2:** Differences in estimated BMI change after diabetes diagnosis

Variable	*n*	Univariable analysis	Multivariable analysis
Sex			
Female	1181	−0.10 (−0.15, −0.05)	−0.08 (−0.13, −0.03)
Male	1555	Reference	Reference
Age (years)			
<50	202	0.03 (−0.07, 0.13)	0.08 (−0.02, 0.18)
50 to <60	556	0.07 (0.01, 0.14)	0.14 (0.07, 0.21)
60 to <70	986	0.07 (0.02, 0.13)	0.11 (0.05, 0.17)
≥70	992	Reference	Reference
BMI (kg/m^2^)			
25 to <30	955	Reference	Reference
30 to <35	970	−0.07 (−0.13, −0.01)	−0.05 (−0.11, −0.01)
35 to <40	492	−0.15 (−0.22, −0.08)	−0.11 (−0.18, −0.03)
≥40	319	−0.28 (−0.36, −0.20)	−0.21 (−0.30, −0.13)
Social deprivation			
1 (most deprived)	598	−0.06 (−0.14, 0.02)	
2	626	−0.04 (−0.12, 0.04)	
3	502	−0.02 (−0.10, 0.06)	
4	465	−0.01 (−0.10, 0.07)	
5 (least deprived)	485	Reference	
Missing	60	−0.02 (−0.20, 0.16)	
Calendar year			
2003 to 2008	676	0.05 (−0.02, 0.13)	
2009 and 2010	640	0.04 (−0.03, 0.11)	
2011 and 2012	733	0.05 (−0.02, 0.12)	
2013 and 2014	687	Reference	
HbA_1c_ (mmol/mol)			
Q1: <43	639	Reference	Reference
Q2: 43 to <48	661	−0.21 (−0.28, −0.14)	−0.15 (−0.22, −0.08)
Q3: 48 to <54	769	−0.33 (−0.40, −0.26)	−0.24 (−0.31, −0.17)
Q4: ≥54	667	−0.31 (−0.38, −0.24)	−0.22 (−0.30, −0.13)
HbA_1c_ (%)			
Q1: <6.1	639	Reference	Reference
Q2: 6.1 to <6.5	661	−0.21 (−0.28, −0.14)	−0.15 (−0.22, −0.08)
Q3: 6.5 to <7.1	769	−0.33 (−0.40, −0.26)	−0.24 (−0.31, −0.17)
Q4: ≥7.1	667	−0.31 (−0.38, −0.24)	−0.22 (−0.30, −0.13)
Pre-diagnosis trajectory			
Rapid gain	875	Reference	
Moderate gain	398	0.01 (−0.07, 0.09)	
Slow gain	463	−0.02 (−0.09, 0.06)	
Stable	374	−0.01 (−0.09, 0.07)	
Slow loss	263	−0.06 (−0.15, 0.03)	
Moderate loss	168	−0.08 (−0.19, 0.03)	
Rapid loss	195	−0.09 (−0.20, 0.01)	
Peri-diagnosis weight change			
Rapid gain	468	−0.37 (−0.44, −0.30)	−0.30 (−0.37, −0.22)
Moderate gain	187	−0.33 (−0.43, −0.23)	−0.28 (−0.38, −0.18)
Slow gain	229	−0.22 (−0.32, −0.13)	−0.19 (−0.28, −0.10)
Stable	253	−0.25 (−0.34, −0.16)	−0.22 (−0.31, −0.13)
Slow loss	307	−0.18 (−0.26, −0.09)	−0.15 (−0.24, −0.07)
Moderate loss	306	−0.20 (−0.28, −0.11)	−0.16 (−0.25, −0.08)
Rapid loss	986	Reference	Reference

For the univariable analysis, values are unadjusted coefficients (95% CI). Units are BMI change (kg/m^2^) per year (over the 4-year study period) adjusted for diabetes medications and change in HbA_1c_. For the multivariable analysis, values are adjusted coefficients (95% CI). Units are BMI change (kg/m^2^) per year (over the 4-year study period), adjusted for diabetes medications, change in HbA_1c_, sex, age, BMI and HbA_1c_ at 1 year from diagnosis, and peri-diagnosis weight change. Values are expressed as the difference in BMI change between the study group and the reference group. A positive value indicates weight gain and a negative value indicates weight loss compared with the reference group

In the multivariable model, the univariable associations remained: individuals with a rapid weight gain in the peri-diagnosis period lost 0.30 kg/m^2^ (0.22, 0.37) per year more than those with a rapid weight loss, women lost 0.08 kg/m^2^ (0.03, 0.13) per year more than men, individuals diagnosed between age 50 to less than 60 years gained 0.14 kg/m^2^ (0.07, 0.21) more than those 70 years or older, individuals with BMI ≥ 40 kg/m^2^ lost 0.21 kg/m^2^ (0.13, 0.30) per year more than individuals with BMI <30 kg/m^2^, and individuals in the highest HbA_1c_ quartile (≥54 mmol/mol or ≥7.1%) lost 0.22 kg/m^2^ (0.13, 0.30) per year more than individuals in the lowest HbA_1c_ quartile (<43 mmol/mol or <6.1%).

To further explore the inverse relationship between peri- and post-diagnosis weight change, the overall patterns of HbA_1c_ and BMI during the peri- and post-diagnosis periods are shown in Fig. 2a,b, comparing individuals with rapid weight loss during the peri-diagnosis period (986 individuals; 36% of the study population) to those with rapid weight gain (468 individuals; 17% of the study population). In addition, patient characteristics during the study period, divided by peri-diagnosis weight change category, are presented in ESM Table 4. In the rapid weight loss group, HbA_1c_ is significantly elevated during the peri-diagnosis period (56.9±18.7 vs 52.3±15.9 mmol/mol in the rapid gain group [7.4±1.7 vs 6.9±1.5%]; means ± SD), and this group are most likely to be treated with drugs around diagnosis (38% compared with 28% in the rapid gain group). However, 1 year after diagnosis, this group have the lowest HbA_1c_ (49.2±14.3 vs 52.8±12.6 mmol/mol in the rapid gain group [6.7±1.3 vs 7.0±1.2%]), and this remains the case at 5 years (52.1±13.3 vs 54.8±15.1 mmol/mol in the rapid gain group [6.9±1.2 vs 7.2±1.4%]). Furthermore, after the initial peri-diagnosis weight loss, BMI remained stable over the post-diagnosis study period (32.4±5.5 and 32.4±5.9 kg/m^2^ at 1 and 5 years after diagnosis, respectively).

## Discussion

In this large, observational, population-based study of Scottish individuals with type 2 diabetes, there was a pattern of weight gain pre-diagnosis, followed by a pattern of weight loss after diagnosis. In the period between 3 years and 6 months pre-diagnosis, 71% of individuals gained weight, with 32% gaining more than 0.5 kg/m^2^ per year. Conversely, in the period 1 to 5 years after diagnosis, 63% of individuals lost weight, with 23% losing more than 0.5 kg/m^2^ per year. Younger age and greater social deprivation were strongly associated with increased weight gain before diabetes diagnosis, with older and less socially deprived people gaining very little. Pre-diagnosis weight change was unrelated to post-diagnosis weight change, and post-diagnosis weight loss was associated with older age, female sex, higher BMI, higher HbA_1c_ and weight gain during the 18-month peri-diagnosis period.

In keeping with previously published results, monotherapy with metformin was associated with weight loss after diabetes diagnosis [16], and treatment with sulfonylureas, thiazolidinediones or insulin was associated with weight gain [17]. Although the numbers treated with two or more agents in the post-diagnosis window were small, we observed that metformin in combination with SGLT2i, GLP-1RA or DPP4i was associated with weight loss, but treatment with sulfonylureas combined with DPP4i was associated with weight gain. In addition, weight loss was associated with HbA_1c_ reduction, with those having a ≥10% decrease in HbA_1c_ losing 0.43 kg/m^2^, and those with a ≥10% increase in HbA_1c_ showing a 0.48 kg/m^2^ weight gain, on average. Our data are observational and causal direction cannot be inferred, but our data are consistent with other studies showing that weight loss improves glycaemic control [2, 18], and that treatment with metformin, SGLT2i or GLP-1RA is associated with weight loss, in contrast to treatment with sulfonylureas, thiazolidinediones or insulin [19].

Our observed pattern of individuals gaining weight in the lead up to diagnosis of type 2 diabetes, followed by progressive weight loss after diagnosis, is in accordance with two other studies in which weight change before and after diabetes were analysed together [8, 9]. In line with the results of these studies, in our study, age strongly influenced weight change before diabetes diagnosis, with individuals who were diagnosed when younger gaining a large amount of weight and those who were diagnosed when older gaining very little: BMI_−3_ and BMI_−0.5_ were 37.1 kg/m^2^ (36.4, 37.8) and 37.9 kg/m^2^ (37.2, 38.7), respectively, for those aged <50 years at diagnosis, and 31.7 kg/m^2^ (31.4, 32.0) and 31.8 kg/m^2^ (31.5, 32.2), respectively, for those aged ≥70 years at diagnosis (means and 95% CI). Furthermore, after diagnosis, age-related differences were modest, but older people were more likely to lose weight than younger people: BMI_1_ and BMI_5_ were 37.2 kg/m^2^ (36.4, 37.9) and 36.4 kg/m^2^ (35.7, 37.1), respectively, for those aged <50 years at diagnosis, and 30.9 kg/m^2^ (30.6, 31.3) and 30.1 kg/m^2^ (29.8, 30.5), respectively, for those aged ≥70 years at diagnosis). Additionally, our finding that post-diagnosis weight loss was associated with female sex and higher BMI at diagnosis was in accordance with the results of a larger study using the national Scottish Diabetes Register [12].

Our finding of a strong inverse relationship between peri- and post-diagnosis weight change is interesting. It may partly be explained by regression to the mean. However, de Fine Olivarius et al observed a similar relationship [8], with 35% of patients in their study reporting unintentional weight loss at diagnosis lasting less than 6 months, which was associated with increased plasma glucose at the time of diagnosis. In our study, we have no way of differentiating between voluntary and involuntary weight loss. However, we did observe a pattern of elevated HbA_1c_ at diagnosis in the 36% of individuals with rapid peri-diagnosis weight loss (represented by the red line in Fig. 2), suggestive of involuntary weight loss due to glycosuria and energy deficit. Despite this initial hyperglycaemia in the peri-diagnosis period, this group goes on to have the best glycaemic control over the 4-year post-diagnosis study period, and requires less diabetes treatment than others (ESM Table 4); this is consistent with a presentation exacerbated by glucose toxicity, whereby weight loss and more aggressive initial treatment results in longer-term benefit. By contrast, in the 17% of individuals who experienced rapid weight gain during the peri-diagnosis period, BMI does not peak until around 18 months after diagnosis (Fig. 2b), and at a significantly higher level than in the rapid weight loss group, at which point they begin to lose weight more gradually, suggestive of a voluntary, clinically managed weight loss.

The observational nature of this study may be viewed as a limitation. The proportion of individuals excluded from the study may seem large: the main limiting factor was availability of BMI measurements before diagnosis, with 41% of the eligible study population having no prior BMI measurements and a further 23% only having one (ESM Fig. 1). In clinical practice, in patients without diabetes, BMI may be more regularly measured in older or more obese individuals. We set an exclusion criterion of BMI <25 kg/m^2^ at 1 year after diagnosis (BMI_1_), as individuals with BMI values in this range would be unlikely to have had regular BMI measurements unless they had a co-existing disease. Unsurprisingly, there were differences in the clinical characteristics of those included in the study vs those who were excluded (ESM Table 1). The study population were older, but after restricting the excluded population to overweight and obese individuals, there was no difference in BMI between the study population and excluded patients. However, there was a difference in progression of diabetes at 1 year after diagnosis, with the study population having a lower HbA_1c_ and fewer diabetes medications. This suggests either that measuring BMI frequently in an at-risk population may assist in diagnosing diabetes earlier or those that have their BMI measured frequently are more engaged with their healthcare providers. In addition, given the increase in obesity and advancements in diabetes treatment over the last few years, the inclusion of individuals diagnosed as far back as 2003 may limit the generalisability of the results of this study. However, we included calendar year as a covariate in our models to minimise this impact.

In modelling our pre- and post-diagnosis trajectories, we assume BMI change is linear. We assessed the overall pattern of BMI measurements during the study period (Fig. 1), and, after removal of measurements in the peri-diagnosis period, a linear trend was a reasonable assumption. In addition, as the study periods were standardised for each individual with respect to the timing of their BMI measurements, the trajectories may be interpreted as an annual rate of change over that specific period and are comparable with the rates used in other studies. Furthermore, linear mixed-effects models have previously been used to model long-term trajectories of within-person changes in BMI [20], and we have previously applied a similar model to derive rates of glycaemic deterioration using repeated measurements of HbA_1c_ [14].

In summary, we have undertaken a comprehensive real-world evaluation of the relationship between BMI, HbA_1c_ and diabetes medication in an 8-year window around the time of diagnosis of type 2 diabetes. Overall, average weight increases before diagnosis and decreases after diagnosis. Post-diagnosis weight reduction was associated with use of metformin, SGLT2i or GLP-1RA and greater HbA_1c_ reduction. However, there were significant differences across the population. We show that younger, more socially deprived individuals gain weight pre-diagnosis, but type 2 diabetes is less associated with weight gain in older individuals, consistent with the presence of other drivers for diabetes aetiology in older adults (e.g. beta cell deficiency and/or sarcopenia) [21]. After diagnosis of diabetes, older women tend to lose more weight than younger men. We identified a substantial group of individuals who show a rapid deterioration in glycaemic control and weight loss around the time of diagnosis who subsequently stabilise and have no significant weight gain, low HbA_1c_ and lower treatment requirements at 5 years post-diagnosis. This finding suggests that a high HbA_1c_ at diagnosis is not inevitably associated with a poor outcome, and aggressive treatment and weight loss around the time of diagnosis can result in good glycaemic and weight outcomes.

## Supplementary Information

Below is the link to the electronic supplementary material.

Supplementary file1 (PDF 165 KB)

## Data Availability

The data that support the findings of this study are not openly available for reasons of sensitivity, but are available from the corresponding author upon reasonable request.

## Abbreviations

DPP4i: Dipeptidyl peptidase 4 inhibitors
GLP-1RA: Glucagon-like peptide 1 receptor agonists
SGLT2i: Sodium–glucose co-transporter 2 inhibitors

## References

[1] Dhana K, Nano J, Ligthart S et al (2016) Obesity and life expectancy with and without diabetes in adults aged 55 years and older in the Netherlands: a prospective cohort study. PLoS Med 13(7):e1002086. 10.1371/journal.pmed.1002086 27433939 10.1371/journal.pmed.1002086 PMC4951120

[2] Gummesson A, Nyman E, Knutsson M, Karpefors M (2017) Effect of weight reduction on glycated haemoglobin in weight loss trials in patients with type 2 diabetes. Diabetes Obes Metab 19(9):1295–1305. 10.1111/dom.12971 28417575 10.1111/dom.12971

[3] Heilbronn LK, Noakes M, Clifton PM (1999) Effect of energy restriction, weight loss, and diet composition on plasma lipids and glucose in patients with type 2 diabetes. Diabetes Care 22(6):889–895. 10.2337/diacare.22.6.889 10372237 10.2337/diacare.22.6.889

[4] Keenan PS (2009) Smoking and weight change after new health diagnoses in older adults. Arch Intern Med 169(3):237–242. 10.1001/archinternmed.2008.557 19204214 10.1001/archinternmed.2008.557 PMC3752594

[5] Brancati FL, Wang NY, Mead LA, Liang KY, Klag MJ (1999) Body weight patterns from 20 to 49 years of age and subsequent risk for diabetes mellitus: the Johns Hopkins Precursors Study. Arch Intern Med 159(9):957–963. 10.1001/archinte.159.9.957 10326937 10.1001/archinte.159.9.957

[6] Ford ES, Williamson DF, Liu S (1997) Weight change and diabetes incidence: findings from a national cohort of US adults. Am J Epidemiol 146(3):214–222. 10.1093/oxfordjournals.aje.a009256 9247005 10.1093/oxfordjournals.aje.a009256

[7] Hanson RL, Narayan KMV, McCance DR et al (1995) Rate of weight gain, weight fluctuation, and incidence of NIDDM. Diabetes 44(3):261–266. 10.2337/diab.44.3.261 7883111 10.2337/diab.44.3.261

[8] de Fine Olivarius N, Siersma VD, Køster-Rasmussen R, Heitmann BL, Waldorff FB (2015) Weight changes following the diagnosis of type 2 diabetes: the impact of recent and past weight history before diagnosis. Results from the Danish Diabetes Care in General Practice (DCGP) study. PLoS One 10(4):e0122219. 10.1371/journal.pone.0122219 25876061 10.1371/journal.pone.0122219 PMC4398495

[9] Looker HC, Knowler WC, Hanson RL (2001) Changes in BMI and weight before and after the development of type 2 diabetes. Diabetes Care 24(11):1917–1922. 10.2337/diacare.24.11.1917 11679457 10.2337/diacare.24.11.1917

[10] Nano J, Dhana K, Asllanaj E et al (2020) Trajectories of BMI before diagnosis of type 2 diabetes: the Rotterdam study. Obesity (Silver Spring) 28(6):1149–1156. 10.1002/oby.22802 32379398 10.1002/oby.22802 PMC7317538

[11] Vistisen D, Witte DR, Tabák AG et al (2014) Patterns of obesity development before the diagnosis of type 2 diabetes: the Whitehall II cohort study. PLoS Med 11(2):e1001602. 10.1371/journal.pmed.1001602 24523667 10.1371/journal.pmed.1001602 PMC3921118

[12] Aucott LS, Philip S, Avenell A, Afolabi E, Sattar N, Wild S (2016) Patterns of weight change after the diagnosis of type 2 diabetes in Scotland and their relationship with glycaemic control, mortality and cardiovascular outcomes: a retrospective cohort study. BMJ Open 6(7):e010836. 10.1136/bmjopen-2015-010836 27466237 10.1136/bmjopen-2015-010836 PMC4964186

[13] Morgan CL, Jenkins-Jones S, Evans M, Barnett AH, Poole CD, Currie CJ (2012) Weight change in people with type 2 diabetes: secular trends and the impact of alternative antihyperglycaemic drugs. Diabetes Obes Metab 14(5):424–432. 10.1111/j.1463-1326.2011.01552.x 22192841 10.1111/j.1463-1326.2011.01552.x

[14] Donnelly LA, Zhou K, Doney ASF, Jennison C, Franks PW, Pearson ER (2018) Rates of glycaemic deterioration in a real-world population with type 2 diabetes. Diabetologia 61(3):607–615. 10.1007/s00125-017-4519-5 29260253 10.1007/s00125-017-4519-5 PMC6448965

[15] Scotland’s Census 2022 (2024) Available from https://www.scotlandscensus.gov.uk/2022-results/scotland-s-census-2022-ethnic-group-national-identity-language-and-religion/. Accessed 15 June 2024

[16] Yerevanian A, Soukas AA (2019) Metformin: mechanisms in human obesity and weight loss. Curr Obes Rep 8(2):156–164. 10.1007/s13679-019-00335-3 30874963 10.1007/s13679-019-00335-3 PMC6520185

[17] Apovian CM, Okemah J, O’Neil PM (2019) Body weight considerations in the management of type 2 diabetes. Adv Ther 36(1):44–58. 10.1007/s12325-018-0824-8 30465123 10.1007/s12325-018-0824-8 PMC6318231

[18] Shinde S, Thieu V, Kwan A, Houghton KF, Schapiro D, Meyers J (2022) The relationship between weight loss and HbA1c in people with type 2 diabetes. Diabetes 71(Suppl 1):952-P. 10.2337/db22-952-P

[19] Haddad F, Dokmak G, Bader M, Karaman R (2023) A comprehensive review on weight loss associated with anti-diabetic medications. Life (Basel) 13(4):1012. 10.3390/life13041012 37109541 10.3390/life13041012 PMC10144237

[20] Cheng Y-J, Chen Z-G, Wu S-H et al (2021) Body mass index trajectories during mid to late life and risks of mortality and cardiovascular outcomes: results from four prospective cohorts. eClinicalMedicine 33:100790. 10.1016/j.eclinm.2021.100790 33778436 10.1016/j.eclinm.2021.100790 PMC7985466

[21] Bellary S, Kyrou I, Brown JE et al (2021) Type 2 diabetes mellitus in older adults: clinical considerations and management. Nat Rev Endocrinol 17:534–548. 10.1038/s41574-021-00512-2 34172940 10.1038/s41574-021-00512-2

