# Supplementary material for: Trajectories of BMI before and after diagnosis of type 2 diabetes in a real-world population
Source: Diabetologia. 2024 Jul 5;67(10):2236–45. doi: 10.1007/s00125-024-06217-1 (PMC11446948; doi:10.1007/s00125-024-06217-1)
Supplement: Supplementary file 1 — Supplementary file1 (PDF 165 KB) [file 125_2024_6217_MOESM1_ESM.pdf]

ESM Table 1 Comparison of characteristics at one year from diagnosis of individuals included vs. excluded in the study

**Variable**	**Included**	**Excluded**[a]	***p-value***
n	2736	13,122	
**Age at diagnosis (years):**	65.6 (10.1)	61.1 (11.4)	<0.0001[b]
<50	202 (7.4%)	2384 (18.2%)	
50 to <60	556 (20.3%)	3697 (28.2%)	
60 to <70	986 (36.0%)	3966 (30.2%)	
≥70	99 (36.3%)	3075 (23.4%)	
**Sex:**			
Male	1555 (56.8%)	7365 (56.1%)	
Female	1181 (43.2%)	5757 (43.9%)	0.4973[c]
**BMI (kg/m^2):**	33.0 (5.9)	33.2 (5.9)	0.2523[b]
25 to <30	955 (34.9%)	4506 (34.3%)	
30 to <35	970 (35.5%)	4514 (34.4%)	
35 to <40	492 (18.0%)	2474 (18.9%)	
≥40	319 (11.7%)	1627 (12.4%)	
**HbA_{1c} (mmol/mol):**	50.6 (12.5)	53.8 (14.8)	<0.0001[b]
<48	1300 (47.5%)	4863 (37.1%)	
48 to <58	968 (35.4%)	4734 (36.1%)	
58 to <69	271 (9.9%)	1889 (14.4%)	
≥69	197(7.2%)	1636 (12.5%)	
**HbA_{1c} (%):**	6.8 (1.1)	7.1 (1.4)	<0.0001[b]
<6.5	1300 (47.5%)	4863 (37.1%)	
6.5 to <7.5	968 (35.4%)	4734 (36.1%)	
7.5 to <8.5	271 (9.9%)	1889 (14.4%)	
≥8.5	197(7.2%)	1636 (12.5%)	
**Diabetes treatment:**			
Diet	1774 (64.8%)	6577 (50.1%)	
Monotherapy	792 (29.0%)	5104 (38.9%)	
Dual and triple therapy	149 (5.5%)	1245 (9.5%)	
Insulin	21 (0.8%)	196 (1.5%)	<0.0001[c]
**Social deprivation:**			
1 (most deprived)	598 (21.9%)	2651 (20.2%)	
2	626 (22.9%)	2770 (21.1%)	
3	502 (18.4%)	2512 (19.1%)	
4	465 (17.0%)	2563 (19.5%)	
5 (least deprived)	485 (17.7%)	2255 (17.2%)	
Missing	60 (2.2%)	371 (2.8%)	0.1315[d]
**Calendar year at diagnosis:**			
2003 to 2008	676 (24.7%)	4705 (35.9%)	
2009 and 2010	640 (23.4%)	2389 (18.2%)	
2011 and 2012	733 (26.8%)	3068 (23.4%)	
2013 and 2014	687(25.1%)	2960 (22.6%)	<0.0001[d]

Data are mean (SD) or n (%); [a] comparator group are selected from 19,452 (ESM figure 1) with an HbA_{1c} and BMI measurement (restricted to individuals ≥25 kg/m^2) between one and two years from diagnosis; [b]*t* test, [c]X^2 test; [d] X^2 test for trend.

ESM Table 2 Drug effects for most prescribed treatment(s) derived from linear mixed model of BMI trajectories after diagnosis

**Diabetes treatment**	**Estimate (95% CI)**	**Total number of BMI measurements (*n* (%))**
Untreated (diet treated)	Reference	10670 (53.7%)
Insulin (including in combination with other diabetes treatment(s))	**0.29 (0.01, 0.58)**	309 (1.6%)
**Monotherapy:**		
Metformin	**-0.32 (-0.40, -0.24)**	5800 (29.2%)
SU	**0.31 (0.14, 0.48)**	704 (3.5%)
DPP4i	-0.34 (-0.69, 0.02)	92 (0.5%)
TZD	**1.22 (0.76, 1.68)**	53 (0.3%)
**Dual therapy:**		
Metformin and SU	-0.01 (-0.15, 0.12)	1006 (5.1%)
Metformin and DPP4i	**-0.39 (-0.59, -0.19)**	280 (1.4%)
Metformin and TZD	0.24 (-0.07, 0.55)	180 (0.9%)
Metformin and GLP-1RA	**-0.79 (-1.24, -0.34)**	88 (0.4%)
Metformin and SGLT2i	**-1.04 (-1.37, -0.71)**	87 (0.4%)
SU and DPP4i	**0.61 (0.18, 1.05)**	85 (0.4%)
**Triple therapy:**		
Metformin and SU and DPP4i	-0.001 (-0.27, 0.27)	182 (0.9%)
Metformin and SU and TZD	**0.72 (0.29, 1.15)**	86 (0.4%)
Metformin and SU and GLP-1RA	-0.29 (-0.74, 0.16)	58 (0.3%)

SU: Sulphonylureas; TZD: Thiazolidinediones; DPP4i: Dipeptidyl Peptidase-4 Inhibitors; GLP-1RA: glucagon-like peptide agonists; SGLT2i: sodium-glucose co-transporter-2 Inhibitors; The coefficient presented in the table is the association of the drug (s) with BMI (kg/m^2), with untreated measurements as the reference group

ESM Table 3 %HbA_{1c} change derived from linear mixed model of BMI trajectories after diagnosis

HbA_{1c} change category	Estimate (95% CI)	Total number of BMI measurements *n*(%)
≥10% decrease	-0.43 (-0.50, -0.36)	2777 (14.0%)
>0 to <10% decrease	-0.14 (-0.19, -0.09)	3232 (16.3%)
No change	Reference	4212 (21.2%)
>0 to <10% increase	0.25 (0.21, 0.30)	4366 (22.0%)
≥10% increase	0.48 (0.42, 0.53)	5279 (26.6%)

The coefficient presented in the table is the association of the %HbA_{1c} change with BMI (kg/m^2), with no change in HbA_{1c} as the reference group.

ESM Table 4 Characteristics of individuals split by peri-diagnosis weight change category

Variable	Rapid loss	Medium loss	Slow loss	stable	Slow gain	Medium gain	Rapid gain	*p-value*[a]
n	986 (36%)	306 (11.2%)	307(11.2%)	253(9.3%)	229(8.4%)	187(6.8%)	468(17.1%)	
Age at diagnosis (years)	65.7(10.6)	67.5(9.7)	66.2(9.3)	65.8(9.5)	65.4(10.3)	65.7(10.0)	63.8(9.8)	0.0007
% Female	48.8%	39.9%	35.2%	40.7%	35.8%	40.1%	44.9%	0.1629
BMI_{-3} (kg/m^2)	34.4(5.7)	32.4(5.3)	32.2(5.2)	31.8(4.7)	32.3(5.3)	32.4(5.5)	33.7(5.8)	0.0248
$BMI_{-0.5}$ (kg/m^2)	35.2(5.9)	33.0(5.3)	32.8(5.4)	32.2(4.9)	32.4(5.3)	32.5(5.7)	33.6(6.1)	<0.0001
BMI_1 (kg/m^2)[c]	32.4(5.5)	32.0(5.4)	32.3(5.4)	32.2(4.9)	32.8(5.4)	33.4(5.7)	35.9(6.3)	<0.0001
BMI_5 (kg/m^2)[d]	32.4(5.9)	31.3(5.2)	31.7(5.4(	31.3(4.8)	32.0(5.7)	32.2(5.7)	34.5(6.5)	<0.0001
HbA_{1cdiag} (mmol/mol)[b]	56.9(18.7)	55.6(17.5)	53.2(14.8)	51.8(14.7)	52.7(12.9)	51.4(12.7)	52.3(15.9)	<0.0001
HbA_{1c1} (mmol/mol)[c]	49.2(14.3)	51.3(12.2)	50.3(10.1)	50.1(10.5)	51.4(10.4)	50.8(9.8)	52.8(12.6)	<0.0001
HbA_{1c5} (mmol/mol)[d]	52.1(13.3)	53.1(13)	54.0(13)	53.9(13.7)	54.9(13.6)	54.0(12.8)	54.8(15.1)	0.0007
HbA_{1cdiag} (%)[b]	7.4(1.7)	7.2(1.6)	7.0(1.4)	6.9(1.3)	7.0(1.2)	6.9(1.2)	6.9(1.5)	<0.0001
HbA_{1c1} (%)[c]	6.7(1.3)	6.8(1.1)	6.8(0.9)	6.7(1.0)	6.9(1.0)	6.8(0.9)	7.0(1.2)	<0.0001
HbA_{1c5} (%)[d]	6.9(1.2)	7.0(1.2)	7.1(1.2)	7.1(1.3)	7.2(1.2)	7.1(1.2)	7.2(1.4)	0.0007
**Diabetes $treatment_{diag}$[e]:**								
Diet	61.5%	65.4%	64.5%	72.7%	70.3%	73.8%	72.2%	
Monotherapy	31.2%	27.8%	30%	22.1%	23.1%	23%	20.7%	
Dual and triple therapy	6.3%	5.2%	5.5%	4.4%	5.2%	2.7%	6.2%	
Insulin	1%	1.6%	0%	0.8%	1.3%	0.5%	0.9%	
**Diabetes $treatment_1$[f]:**								
Diet	60.8%	65.4%	63.5%	69.6%	68.1%	68.1%	67.3%	
Monotherapy	32.6%	28.4%	31.6%	26.1%	24.9%	24.9%	24.8%	
Dual and triple therapy	5.9%	5.2%	4.9%	3.6%	5.7%	5.7%	7.1%	
Insulin	0.8%	1%	0%	0.8%	1.3%	1.3%	0.9%	
**Diabetes $treatment_5$[g]:**								
Diet	48.8%	50.3%	42.7%	50.2%	37.1%	47.6%	39.2%	
Monotherapy	32.3%	31.1%	37.1%	33.6%	41.9%	40.6%	35.3%	
Dual and triple therapy	16.9%	16.3%	19.2%	14.6%	17.5%	10.2%	21.8%	

Insulin	2%	2.3%	1%	1.6%	3.5%	1.6%	3.6%	

Data are mean(SD) or *n*(%); [a]Rapid loss vs. rapid gain, *t*-test for continuous variables and X^2-test for categorical; [b] closest to diagnosis but within peri-diagnosis window; [c] first measurement after one year from diagnosis (in 1 to 2 year from diagnosis window); [d] closest to 5 years after diagnosis (in 4 to 5 years from diagnosis window); [e]drugs in the peri-diagnosis window; [f]drugs between 1 year and 18 months from diagnosis; [g]drugs between 4.5 and 5 years after diagnosis.

ESM Figure 1 Study population derivation

22,517
Type 2 diabetes
Diagnosed 2003 to 2014
Complete EMR coverage from diagnosis

↓ → **- 454** Age at diagnosis <35
- 2611 Left area or died within 5 years of diagnosis

19,452
≥5 years follow up from diagnosis

↓ → **- 8063** no prior BMI measurements
- 4497 only one prior BMI measurement
- 3486 2 or more prior measurements but not within required time windows (defined as -3 to -2 and -1.5 to -0.5 years)

3406
Minimum data requirement for trajectory before diagnosis

↓ → **- 52** with BMI data for pre but not for post trajectory

3354
Minimum data requirement for trajectory after diagnosis

↓ → **- 279** not overweight/obese at 1 year from diagnosis

307
$BMI_1 \geq 25$ kg/m^2

↓ → **- 242** with number of BMI measurements in pre or post trajectory above 95% quantile (defined as >8 and >14 respectively)

2833
Individuals with extreme number of BMI measurements removed

↓ → **- 16** with diabetes medications between -3 and -0.5 years pre-diagnosis

2817
No diabetes medications during BMI trajectory before diagnosis

↓ → **-81** with missing HbA1c at 1 year from diagnosis

2736
With HbA_{1c} measured at 1 year from diagnosis

↓

FINAL STUDY POPULATION = 2736
